# Early diagnosis and treatment of Leukocytoclastic Vasculitis: case report

**DOI:** 10.1590/1677-5449.190072

**Published:** 2020-01-07

**Authors:** Alexandre Sacchetti Bezerra, Afonso César Polimanti, Rafael Andretti de Oliveira, Rafael Vilhena de Carvalho Fürst, Paulo Ricardo Criado, João Antônio Corrêa

**Affiliations:** 1 Faculdade de Medicina do ABC, Santo André, SP, Brasil.; 2 Sociedade Brasileira de Angiologia e Cirurgia Vascular – SBACV, São Paulo, SP, Brasil.; 3 Universidade de São Paulo – USP, Faculdade de Medicina, São Paulo, SP, Brasil.

**Keywords:** leukocytoclastic, cutaneous, algorithms, diagnosis, vasculitis

## Abstract

A 46-year-old female patient presented at the emergency department of a Municipal University Hospital with necrotic lesions in lower limbs associated with wasting syndrome. She was diagnosed with leukocytoclastic vasculitis after physical examination and history-taking in a fast and cost-effective manner, using an algorithm specifically for primary vasculitis, enabling early and appropriate treatment. The good clinical outcome demonstrates the need to quickly make a definitive diagnosis and start treatment.

## INTRODUCTION

Cutaneous leukocytoclastic vasculitis (CLV), also known as hypersensitivity vasculitis, is a vasculitis of small vessels that primarily involves the venules and has pathophysiology associated with deposition of immune complexes and characteristic histopathological findings.^1^^-^ 3

Although involvement is limited to the skin in countless cases, systemic involvement occurs in around 50% of cases, with wasting syndrome and damage to kidneys, joints, lungs, muscles, the heart, gastrointestinal organs, and neurological systems.^2^^,^ 4

In the majority of cases, etiology is idiopathic, but in some cases it is described as secondary to medications, infections, lymphoproliferative disorders, cancer, connective tissue diseases, and inflammatory diseases.^2^^,^ 5

Initially, the lesions appear as localized erythema and macular purpura or urticarial papules which progress to palpable purpura, generally symmetrical and on lower limbs. Rarely, the facial, palmar, and plantar regions and mucosas may be affected.

The lesions can proceed to formation of vesicles, nodules, ulcers, or necrosis measuring from 1 mm to 4 cm and can be asymptomatic or painful.^6^

Differential diagnosis must rule out many different entities, because of the nonspecific and complex clinical presentation, making investigation difficult, slow, and expensive. Use of a systematic workup process is very important to hasten the start of treatment, improving prognosis.^1^^,^ 7

Diagnosis is confirmed by histopathology, with findings of inflammatory infiltrate and fragmentation of the nuclei of neutrophils in the vascular walls, which is known as karyorrhexis and is related to fibrinoid necrosis.^3^^,^ 8

This report describes a case of CLV in which a systematic algorithm was used for diagnostic investigation, enabling early treatment with good results.^7^

## CASE DESCRIPTION

E.H.F, a 46-year-old woman, sought urgent medical care complaining of high intensity pain, low temperature, hyperemia, and paresthesia in both feet, with onset 3 months previously. She also described deterioration of visual acuity, migratory polyarthralgia, and weight loss of 12 kg over the previous 2 years. She also reported history of Hashimoto’s thyroiditis and pulmonary tuberculosis, treated 21 years earlier.

**Figure 1:**
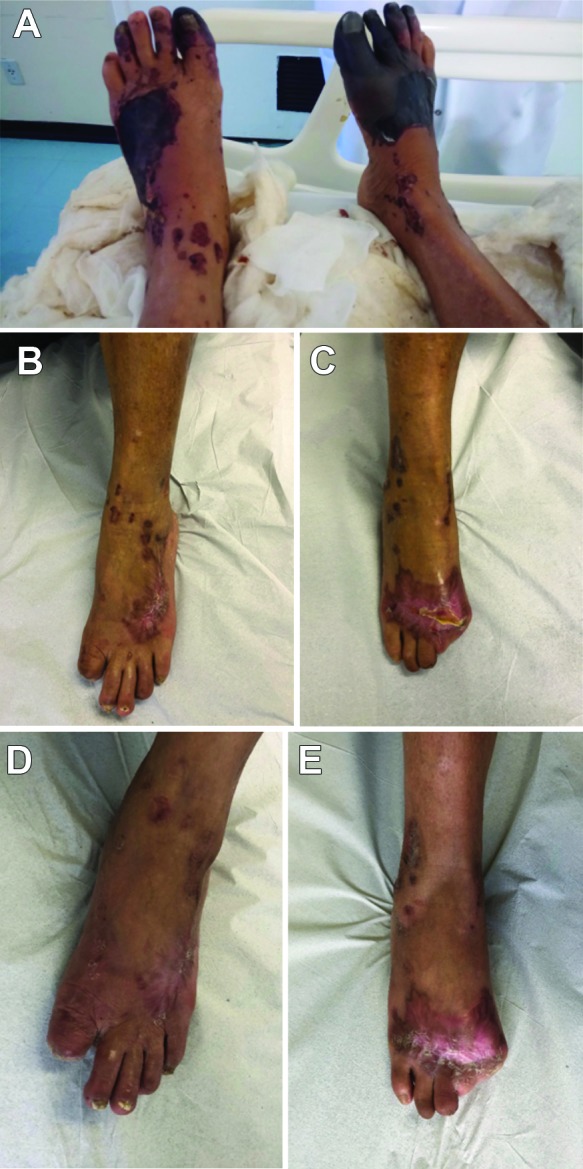
(A) Physical examination on 27 September, 2017; (B) physical examination on 29 December, 2018; (C) physical examination on 29 December, 2018; (D) physical examination on 26 February, 2019; (E) physical examination on 26 February, 2019.

On physical examination, both of the patient’s lower limbs were cyanotic with blisters (as shown in Figure 1A), but pedal pulses were present and symmetrical. In view of the weight loss associated with cutaneous lesions described above, a diagnostic hypothesis was ventured of primary small vessel vasculitis.

**Figure 2:**
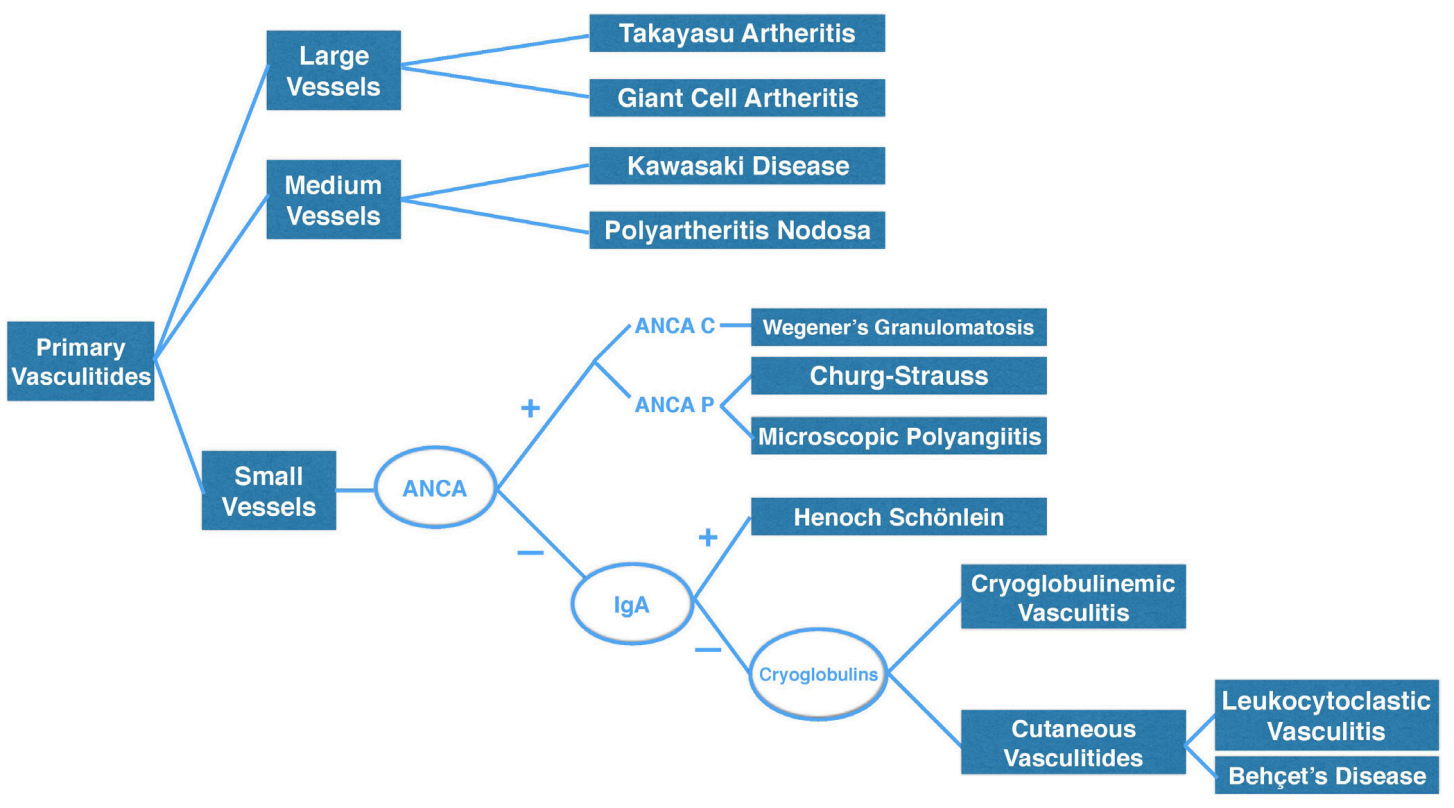
The Bezerra algorithm.^7^

The Bezerra algorithm was consulted (illustrated in Figure 2), observing that ANCA P, ANCA C, cryoglobulins, and IgA were all negative, suggesting a diagnosis of CLV.^7^

After definition of this diagnosis, on 29 September, 2017, amputations were performed of both halluxes and the distal phalanx of the 2nd, 3rd, and 4th toes of the right foot, ordering pathology of the tissues amputated from the feet, which confirmed the diagnosis of CLV, by “fragmentation of neutrophil nuclei”.

Treatment was initiated with methylprednisolone, 20 mg every 6 h for 3 days, cilostazol 100 mg every 12 h, and cyclophosphamide monthly, in a single 750 mg dose. Figures 11C illustrate the patient’s clinical status after 3 months’ treatment, with surgical wounds healed; during which time she had partially recovered the ability to walk. After 6 months of treatment and clinical follow-up, the patient had completely recovered the ability to walk.

Figures 11E illustrate the anatomic appearance of the lesions after stabilization of clinical status. Currently, after 19 months, the patient is in outpatients follow-up at the vascular surgery service and the rheumatology service, taking prednisone 20 mg a day, cilostazol 100 mg every 12 h, and cyclophosphamide in a single 750 mg dose once a month.

## DISCUSSION

The pathogenic mechanism of CLV is deposition of immune complexes, generally IgG and IgM immunoglobulins, which activate the complement cascade, with production of factors chemotactic for leukocytes and expression of adhesion molecules.^2^^,^ 9

As a result, neutrophils migrate to the area, releasing enzymes and reactive oxygen species to eliminate antigens. However, the reactive inflammatory process is intense and damages the vessel walls, increasing the permeability of these vessels, with leakage of fluids and red blood cells. This process also damages neutrophil cells, which releases inflammatory substances.^4^^,^ 5 ^,^ 8 ^,^ 9

Epidemiologically, there is no uniform pattern of either the prevalence or the incidence of this condition or the other primary vasculitides reported in published studies.^10^

Tai et al. observed a prevalence of 73.2% of patients with CLV in a sample of 93 patients.^11^ In a study by Blanco et al., with 303 patients, 27.7% had CLV.^12^ Jokar and Mirfeizi cataloged 721 patients, finding CLV in 8.2% deles.^13^ Clinically, the painful symptoms associated with cutaneous manifestations play an imperative role in formulation of a diagnostic hypothesis.

In a study by Sais et al., cutaneous eruptions were observed in 41.4% of patients. Arthralgia was the most common complaint, seen in 36.7% of the patients.^14^ In cases limited to cutaneous involvement, the duration of the disease is generally short if appropriate treatment is instituted. Purpural lesions can recede in 3 to 4 weeks.^15^ Although it is self-limiting in the majority of cases, CLV can relapse months or years later.

Supplementary tests such as blood counts, angiotomography, inflammatory activity tests, colonoscopy, serum complement levels, serology, ANF, rheumatoid factor, antiphospholipid antibodies, and urine analysis are expensive and nonspecific and are only of utility for assessing organ function. In addition to their low specificity and high cost, these tests can also delay definitive diagnosis.^7^^,^ 16

The American College of Rheumatology consensus bases diagnosis of CLV on the following criteria:

- Patient over the age of 16 years at disease onset;
- Use of medication and its correlation with onset of the disease;
- Palpable purpura;
- Maculopapular Exanthema;
- Histopathological study encompassing arterioles and venules with presence of perivascular or extravascular granulocytes.

Presence of three of the five items listed has specificity of 83.9% and sensitivity of 71%.^1^^,^ 17

Use of the algorithm mentioned above was of fundamental importance to making a rapid and economically feasible diagnosis.^7^

Cutaneous biopsy may be indispensable to diagnosis of this disease. In addition to assessing the caliber of the vessels involved, pathology will also evaluate the possibility of other diagnostic hypotheses such as perivasculitis, embolic phenomena, and other non-inflammatory vasculopathies that simulate vasculitis.

Microscopic analysis should be performed during the initial phase of the inflammatory process, since older lesions may no longer show the characteristics typical of leukocytoclasia.^18^^,^ 19

Histopathological examination will reveal an angiocentric inflammatory process, associated with leukocytoclasia, characterized by fragmentation of neutrophil nuclei, edema of endothelial cells, leakage of red blood cells, and fibrinoid necrosis. It may be possible to observe the presence of immunoglobulins in these lesions using immunofluorescence, but they are present in low quantities in vascular lesions and their value for routine testing is debatable.^3^^,^ 9 ^,^ 20 Unfortunately, this case report is limited by not having photographic images of the slide examined.

The standard treatment currently employed to achieve remission is prednisone 1 mg/kg/day with cyclophosphamide 2 mg/kg/day; generally maintained for 6 months. Rituximab is not available at our service, but some other services use it to treat this disease.^21^

The countless publications on vasculitis in the literature are unanimous on the high complexity of etiologic investigation, causing excessive delays, elevated costs and delayed treatment.^1^^,^ 7

The case of CLV described here illustrates how treatment success is dependent on rapid diagnostic investigation and early treatment. In this context, it is evident how important it is to employ our algorithm to avoid delaying institution of immunosuppressant therapy.
