# Supplementary material for: Early diagnosis and treatment of Leukocytoclastic Vasculitis: case report
Source: J Vasc Bras. 2020 Jan 7;19:e20180072. doi: 10.1590/1677-5449.190072 (PMC6956624; doi:10.1590/1677-5449.190072)
Supplement: Translation

# Translation: Diagnóstico e terapêutica precoce de vasculite leucocitoclástica: relato de caso

**Authors:** Alexandre Sacchetti Bezerra, Afonso César Polimanti, Rafael Andretti de Oliveira, Rafael Vilhena de Carvalho Fürst, Paulo Ricardo Criado, João Antônio Corrêa

**Affiliations:** 1 Faculdade de Medicina do ABC, Santo André, SP, Brasil.; 2 Sociedade Brasileira de Angiologia e Cirurgia Vascular – SBACV, São Paulo, SP, Brasil.; 3 Universidade de São Paulo – USP, Faculdade de Medicina, São Paulo, SP, Brasil.

**Keywords:** leucocitoclasia, cutânea, algoritmos, diagnóstico, vasculite

## Abstract

Paciente do sexo feminino, 46 anos de idade, procurou o pronto socorro do Hospital Municipal Universitário apresentando lesões necróticas em membros inferiores associadas à síndrome consumptiva. Após anamnese e exame físico, obteve-se o diagnóstico de vasculite leucocitoclástica de modo imediato e econômico por meio da utilização de algoritmo específico de vasculites primárias, permitindo início precoce da terapêutica adequada. A boa evolução do quadro clínico ratificou a necessidade de se obter diagnóstico definitivo e início rápido da terapêutica.

## INTRODUÇÃO

A vasculite leucocitoclástica cutânea (VLC), conhecida também como vasculite de hipersensibilidade, é uma vasculite de pequenos vasos que acomete principalmente as vênulas, cuja fisiopatologia está associada à deposição de imunocomplexos, com análise histopatológica característica^1^^-^ 3.

Embora, em inúmeros casos, o acometimento seja restrito ao território cutâneo, o envolvimento sistêmico ocorre em cerca de 50% dos casos, com síndrome consumptiva associada a comprometimento renal, articular, pulmonar, muscular, cardíaco, gastrointestinal ou neurológico^2^^,^ 4.

Majoritariamente, tem etiologia idiopática, porém, em alguns casos, é descrita como secundária a medicamentos, infecções, distúrbios linfoproliferativos, neoplasias, doenças do tecido conjuntivo e doenças inflamatórias^2^^,^ 5.

A princípio, as lesões surgem na forma de eritemas localizados e de púrpuras maculares ou pápulas urticariformes que progridem para púrpuras palpáveis, geralmente simétricas em membros inferiores. Raramente acomete as regiões facial, palmar, plantar e mucosas.

Essas lesões podem evoluir para a formação de vesículas, nódulos, úlceras ou necrose de 1 mm a 4 cm, sendo tanto assintomáticas como dolorosas^6^.

Em virtude da inespecificidade e complexidade do quadro clínico, há uma ampla variedade de diagnósticos diferenciais, tornando a investigação difícil, lenta e onerosa. A utilização de um processo sistemático na rotina propedêutica é de fundamental importância, a fim de abreviar o início da terapêutica, permitindo melhor prognóstico^1^^,^ 7.

O diagnóstico é confirmado pelo exame histopatológico, onde se observa infiltrado inflamatório com fragmentação dos núcleos de neutrófilos localizados na parede vascular denominado cariorrexe e associado a necrose fibrinoide^3^^,^ 8.

O relato de caso em questão refere-se à VLC, no qual houve aplicação de algoritmo sistemático na investigação diagnóstica, permitindo tratamento precoce com boa evolução^7^.

## DESCRIÇÃO DO CASO

E.H.F, sexo feminino, 46 anos de idade, procurou o serviço médico de urgência com queixa de dor de forte intensidade associado a diminuição da temperatura, hiperemia e parestesia em ambos os pés há 3 meses. Referia diminuição da acuidade visual, poliartralgia migratória e perda ponderal de 12 kg nos últimos 2 anos. Relatou antecedentes de tireoidite de Hashimoto e tuberculose pulmonar tratada há 21 anos.

Ao exame físico, a paciente apresentava ambos os membros com cianose fixa e flictenas, porém com pulsos pediosos presentes e simétricos conforme ilustrado na Figura 1A. Em virtude da perda ponderal associada às lesões cutâneas já descritas, foi feita hipótese diagnóstica de vasculite primária de pequenos vasos.

**Figura 1:** (A) Exame físico em 27/09/2017; (B) exame físico em 29/12/2018; (C) exame físico em 29/12/2018; (D) exame físico em 26/02/2019; (E) exame físico em 26/02/2019.

Optou-se por aplicar o Algoritmo de Bezerra, descrito na Figura 2, em que se observou P-ANCA, C-ANCA, crioglobulinas e IgA negativos, sugerindo hipótese diagnóstica de VLC^7^.

**Figura 2:** Algoritmo de Bezerra^7^.

Após a definição diagnóstica, em 29/09/2017, foi realizada amputação de hálux bilateral e falange distal de 2º, 3º e 4º pododáctilos direito e solicitado exame anátomo patológico do tecido amputado em pés que confirmou o diagnóstico de VLC, por meio da “fragmentação de núcleos de neutrófilos”.

Iniciado tratamento com metilprednisolona, 20 mg de 6 em 6 h, por 3 dias, cilostazol 100 mg de 12 em 12 h e ciclofosfamida 750 mg mensal em dose única. As Figuras 11C apresentam o estado clínico após 3 meses de tratamento com feridas operatórias cicatrizadas; período no qual houve recuperação parcial da deambulação. Com 6 meses de tratamento e seguimento clínico, a paciente retomou a marcha em sua totalidade.

As Figuras 11E denotam a imagem anatômica das lesões após estabilização do quadro clínico. Atualmente, após 19 meses, a paciente está em acompanhamento ambulatorial no serviço de cirurgia vascular e no serviço de reumatologia, em uso de prednisona 20 mg ao dia, cilostazol 100 mg de 12 em 12 h e ciclofosfamida 750 mg mensal em dose única.

## DISCUSSÃO

Na VLC, a deposição de imunocomplexos constitui seu mecanismo patogênico, geralmente envolvendo imunoglobulinas IgG e IgM, que geram a ativação da cascata do complemento, com produção de fatores quimiotáticos para leucócitos e expressão de moléculas de adesão^2^^,^ 9.

Dessa maneira, os neutrófilos migram para a região, liberando enzimas e derivados reativos do oxigênio para eliminar antígenos. No entanto, o processo inflamatório reacional é intenso e causa lesão na parede vascular, aumentando a permeabilidade desses vasos, com saída de fluidos e extravasamento de hemácias. Esse processo também lesa as células neutrofílicas, com subsequente liberação de substâncias inflamatórias^4^^,^ 5 ^,^ 8 ^,^ 9.

Epidemiologicamente, não há um padrão homogêneo tanto na prevalência quanto na incidência dessa afecção junto às demais vasculites primárias nos estudos já publicados^10^.

Tai et al. obtiveram uma prevalência de 73,2% de pacientes com a presença de VLC em um universo de 93 pacientes^11^. No estudo de Blanco et al., envolvendo 303 pacientes, foram evidenciados 27,7% de VLC^12^. Jokar e Mirfeizi catalogaram 721 pacientes, encontrando VLC em 8,2% deles^13^. Clinicamente, os sintomas álgicos associados às manifestações cutâneas representam um papel imperativo na formulação da hipótese diagnóstica.

No estudo de Sais et al., a erupção cutânea foi encontrada em 41,4% dos pacientes. Artralgia foi a queixa mais frequente, presente em 36,7% dos pacientes^14^. Em casos limitados ao acometimento cutâneo, a duração da doença geralmente é breve se houver terapêutica adequada. As lesões purpúricas podem reduzir em 3 a 4 semanas^15^. Apesar de autolimitada na maioria das situações, a VLC pode recidivar durante meses ou anos.

Exames complementares, como hemograma, angiotomografia, provas de atividade inflamatória, colonoscopia, níveis de complemento sérico, sorologias, FAN, fator reumatoide, anticorpos antifosfolípides e análise da urina são onerosos, não específicos e apenas auxiliam na avaliação das funções orgânicas. Além da baixa especificidade e alto custo, esses exames podem retardar o diagnóstico definitivo^7^^,^ 16.

O diagnóstico de VLC é preconizado pelo Colégio Americano de Reumatologia por meio dos seguintes critérios:

- Idade do paciente superior a 16 anos no surgimento da doença;
- Uso de medicamento e sua correlação com o início da doença;
- Púrpura palpável;
- Exantema maculopapular;
- Estudo histopatológico englobando a avaliação da arteríola e da vênula, denotando a presença de granulócitos peri ou extravasculares.

A presença de três dos cinco itens citados pode apresentar uma especificidade de 83,9% e sensibilidade de 71%^1^^,^ 17.

A utilização do algoritmo citado anteriormente foi de fundamental importância na obtenção de um diagnóstico rápido e economicamente viável^7^.

A biópsia cutânea pode ser imprescindível para o diagnóstico dessa doença. Além de avaliar o calibre do vaso acometido, a análise anátomo patológica pesquisa outras hipóteses como perivasculite, fenômenos embólicos e outras vasculopatias não inflamatórias que simulam vasculite.

A análise microscópica deve ser realizada na fase inicial do processo inflamatório, já que lesões antigas podem não apresentar as características típicas da leucocitoclasia^18^^,^ 19.

No exame histopatológico; observa-se processo inflamatório angiocêntrico, associado a leucocitoclasia, caracterizado por fragmentação de núcleos de neutrófilos, edema de células endoteliais, extravasamento de hemácias e necrose fibrinoide. Nessas lesões, pode ser observada a presença de imunoglobulinas por meio da imunofluorescência, porém a presença delas é escassa nas lesões vasculares, e seu valor como exame de rotina é ainda discutível^3^^,^ 9 ^,^ 20. Infelizmente, este relato de caso teve a limitação de não possuir as imagens fotográficas da lâmina em estudo.

O tratamento padrão atualmente empregado para remissão é prednisona 1 mg/kg/dia com ciclofosfamida 2 mg/kg/dia; geralmente mantido por 6 meses. Apesar de não disponível em nosso serviço, o tratamento com rituximab já é utilizado em alguns serviços no tratamento da doença^21^.

Em inúmeras publicações sobre vasculites, a literatura é consensual na elevada complexidade da investigação etiológica, ocasionando demora excessiva, elevado custo e retardo no início do tratamento^1^^,^ 7.

O caso de VLC relatado denota que o sucesso terapêutico depende de uma investigação diagnóstica rápida com tratamento precoce. Dentro desse contexto, fica evidente a importância do uso do nosso algoritmo para não retardar o início da terapêutica imunossupressora.
